# Process to Remove the Size Variants Contained in the Antibody–Chelator Complex PCTA-NCAB001 for Radiolabeling with Copper-64

**DOI:** 10.3390/ph16101341

**Published:** 2023-09-22

**Authors:** Yukie Yoshii, Hiroki Matsumoto, Chika Igarashi, Tomoko Tachibana, Fukiko Hihara, Mitsuhiro Shinada, Atsuo Waki, Sei Yoshida, Kenichiro Naito, Kimiteru Ito, Tatsuya Higashi, Hiroaki Kurihara, Makoto Ueno

**Affiliations:** 1 Institute for Quantum Medical Science, National Institutes for Quantum Science and Technology, Chiba 263-8555, Japan; matsumoto.hiroki2@qst.go.jp (H.M.); igarashi.chika@qst.go.jp (C.I.); tachibana.tomoko@qst.go.jp (T.T.); hihara.fukiko@qst.go.jp (F.H.); shinada.mitsuhiro@qst.go.jp (M.S.); waki.atsuo@qst.go.jp (A.W.); higashi.tatsuya@qst.go.jp (T.H.); 2 Department of Diagnostic Radiology, Kanagawa Cancer Center, Yokohama 241-8515, Japan; h-kurihara@kcch.jp; 3 Department of Biology, Graduate School of Science, Toho University, Chiba 274-8510, Japan; 4 Department of Chemistry, Graduate School of Science, Toho University, Chiba 274-8510, Japan; 5 Department of Research, NanoCarrier Co., Ltd., Tokyo 104-0031, Japan; yoshida@nanocarrier.co.jp (S.Y.); naito@nanocarrier.co.jp (K.N.); 6 Department of Diagnostic Radiology, National Cancer Center Hospital, Tokyo 104-0045, Japan; kimito@ncc.go.jp; 7 Department of Gastroenterology, Kanagawa Cancer Center, Yokohama 241-8515, Japan; uenom@kcch.jp

**Keywords:** antibody–drug conjugates, antibody–chelator conjugates, chromatography, size exclusion, multi-angle light scattering, hydrophobic interaction chromatography

## Abstract

Understanding the physicochemical properties of antibody–drug conjugates is critical to assess their quality at manufacturing and monitor them during subsequent storage. For radiometal–antibody complexes, it is important to control the properties of the antibody–chelator conjugate to maintain the quality of the final product. We have been developing ^64^Cu-labeled anti-epidermal growth factor receptor antibody NCAB001 (^64^Cu-NCAB001) for the early diagnosis and therapy of pancreatic cancer with positron-emission tomography. Here, we characterized the larger size variants contained in the antibody–chelator conjugate PCTA-NCAB001 by multi-angle light scattering coupled with size-exclusion chromatography. Secondly, we developed a chromatographic method to remove these size variants. Lastly, we demonstrated the stability of PCTA-NCAB001 after the removal of size variants. Dimer and oligomers were identified in PCTA-NCAB001. These larger size variants, together with some smaller size variants, could be removed by hydrophobic interaction chromatography. The PCTA-NCAB001 product, after the removal of these size variants, could be stored at 4 °C for six months. The methods developed here can be applied to assure the quality of PCTA-NCAB001 and other antibody–drug conjugates to facilitate the development of antibody–radiometal conjugates for positron-emission tomography and radioimmunotherapy of malignant cancers.

## 1. Introduction

Monoclonal antibodies conjugated with radiometals are becoming increasingly important tools in medical research and clinical practice. Several types of radiometals are used such as cytotoxins constructing the antibody–drug conjugates (ADCs). For the conjugation of radiometals to antibodies, a suitable combination of radioisotope and bifunctional chelator is important.

Among the radiometals, we chose copper-64 (^64^Cu) since it emits β^+^ particles for imaging with positron-emission tomography (PET) as well as β^−^ particles and Auger electrons for cancer therapy [1]. The half-life of ^64^Cu is suitable for evaluating the pharmacokinetics of antibodies in humans; in addition, it can be produced by a biomedical cyclotron which is widely available in clinical practice [2]. For the labeling of the monoclonal antibody with ^64^Cu, we chose the bifunctional chelator 3,6,9,15-tetraazabicyclo [9.3.1]pentade-ca-1(15),11,13-triene-4-S-(4-isothiocyanatobenzyl)-3,6,9-triacetic acid (p-SCN-Bn-PCTA). We stably labeled the anti-epidermal growth factor receptor (EGFR) antibody cetuximab with ^64^Cu using p-SCN-Bn-PCTA for the imaging and therapy of pancreatic and gastrointestinal cancers [1]. We choose cetuximab for ^64^Cu-labeling since the overexpression of EGFR has been observed in up to 90% of pancreatic cancers [3,4]. In addition, fluorescent dye conjugated cetuximab could be used to detect pancreatic cancer lesions intraoperatively [5]. These findings support that EGFR is a good target for PET imaging and radioimmunotherapy of pancreatic cancer.

Early diagnosis and treatment of pancreatic cancer are critical for obtaining better patient outcomes [6,7,8,9,10]. However, identification of small resectable pancreatic cancer using current imaging modalities remains challenging [11,12,13]. To address this challenge, we developed a new anti-EGFR antibody NCAB001, and labeled it with ^64^Cu using p-SCN-Bn-PCTA (^64^Cu-NCAB001). In an orthotopic xenograft mouse model of small resectable (<1 cm) pancreatic cancer, tumor lesions ≥ 3 mm were clearly identified using PET imaging through intraperitoneal administration of ^64^Cu-NCAB001 [14]. Intraperitoneal administration of ^64^Cu-NCAB001 in non-human primates was well tolerated from the aspects of biodistribution, radiation dosimetry, and pharmacologic profile [14]. An extended single-dose toxicity study of intraperitoneally administered Cu-NCAB001 in mice was conducted according to approach 1 of the current International Conference on Harmonisation of Technical Requirements for Registration of Pharmaceuticals for Human Use M3 [R2] guideline [15]. The no-observed-adverse-effect level of Cu-NCAB001 was 625 μg/kg, which is approximately 1000-fold higher than the dose of ^64^Cu-NCAB001 in our current investigational drug formulation (45 µg) at the μg/kg level [16].

To bring ADCs to the clinical practice, development of the analytical methods to access the binding properties of the antibody, potency of the drugs, drugs to antibody ratio (DAR), in vivo and in vitro stability of the conjugates, and size variants of the conjugates are critical [17]. Since ^64^Cu has a physical half-life of 12.7 h, it is important to understand the physiochemical properties of the antibody–chelator conjugate, PCTA-NCAB001, to maintain the quality of the final product ^64^Cu-NCAB001. In our previous studies, we developed the formulation to stabilize PCTA-NCAB001 [18]. We determined the DAR of PCTA-NCAB001 by liquid chromatography–mass spectrometry (LC–MS). By enzyme-linked immunosorbent assay (ELISA), the binding potency of PCTA-NCAB001 against recombinant human EGFR was confirmed [18]. We also found larger size variants contained in PCTA-NCAB001 by size-exclusion high-performance liquid chromatography (SEC-HPLC) [16].

Further understanding of the physicochemical properties of PCTA-NCAB001 was needed to assess its quality at manufacturing and monitor it during subsequent storage. Here, we characterized the larger size variants contained in PCTA-NCAB001. Secondly, we developed a method to remove these size variants. Lastly, we demonstrated the long-term stability of PCTA-NCAB001 after the removal of these size variants. These results, together with our previous findings, can be used to assess the quality of PCTA-NCAB001 as the final intermediate of ^64^Cu-NCAB001 in future clinical practice.

## 2. Results

### 2.1. Identification of the Size Variants Contained in PCTA-NCAB001

**Figure 1:**
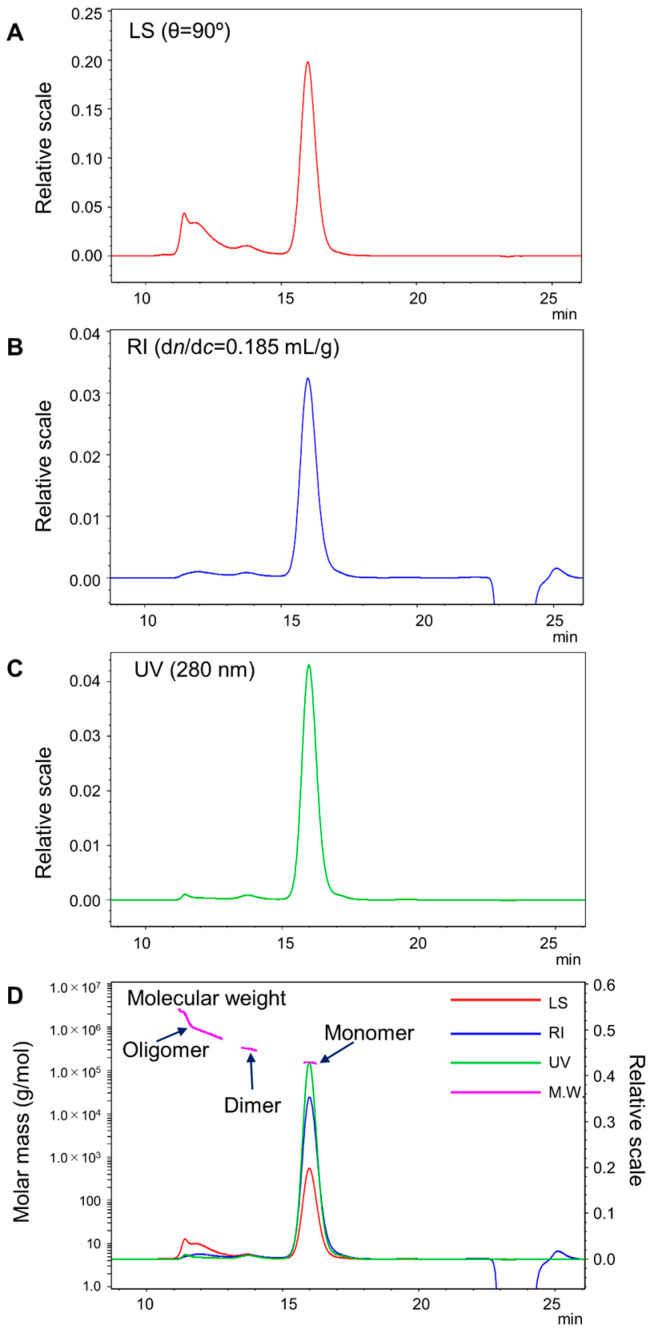
Size-exclusion chromatogram of PCTA-NCAB001. Detections were made by light scattering (LS) (**A**), refractory index (RI) (**B**), and ultraviolet absorption (UV) (**C**). These chromatograms were overlayed, and molecular weights (M.W.) were plotted (**D**). Larger size variants were detected at retention times of 11 min and 13 min.

The anti-EGFR antibody NCAB001 was prepared according to the current good manufacturing practices (cGMP), and the NCAB001 antibody and the bifunctional chelator p-SCN-Bn-PCTA were conjugated as previously reported [14,18]. Bovine serum albumin (BSA, Pierce™ Bovine Serum Albumin Standard Ampules, 2 mg/mL, lot number VJ312468, Thermo Fisher Scientific, Waltham, MA, USA) was measured by multi-angle light scattering coupled with size-exclusion chromatography (SEC-MALS) system and molecular weight was confirmed. Thereafter, PCTA-NCAB001 was measured by SEC-MALS; the chromatograms are shown in Figure 1. *θ* = 90° was selected as the representative chromatogram of the light scattering (LS). Monomers of PCTA-NCAB001 were eluted at a retention time of 16 min. Larger size variants were detected at retention times of 11 min and 13 min. The size variant detected at 13 min had a molecular weight of 3.190 × 10^5^ g/mol, and that detected at 11 min had a molecular weight larger than 5.00 × 10^5^ g/mol (Figure 1D). Molecular weights and multi-variance of PCTA-NCAB001 and size variants are summarized in Table 1. The size variant detected at 13 min was assigned as a dimer, and the size variant detected at 11 min was a complex of oligomers.

### 2.2. Removal of the Size Variants Contained in PCTA-NCAB001

**Figure 2:**
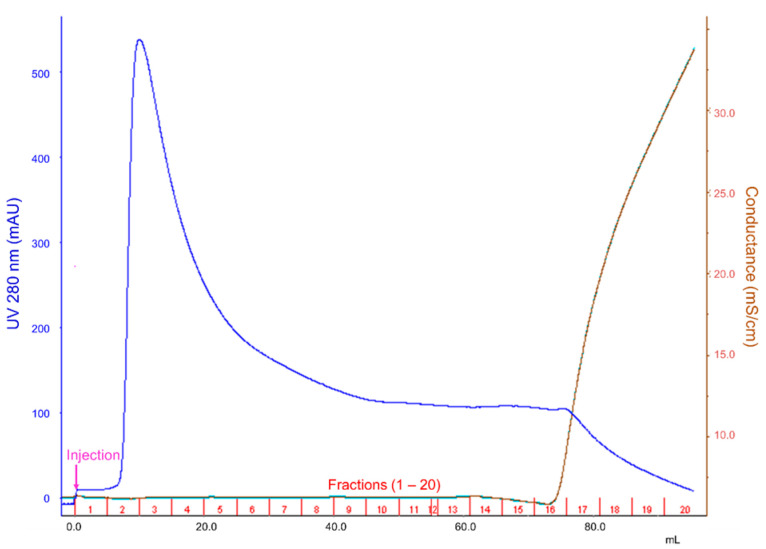
Hydrophobic interaction chromatogram of PCTA-NCAB001.

**Figure 3:**
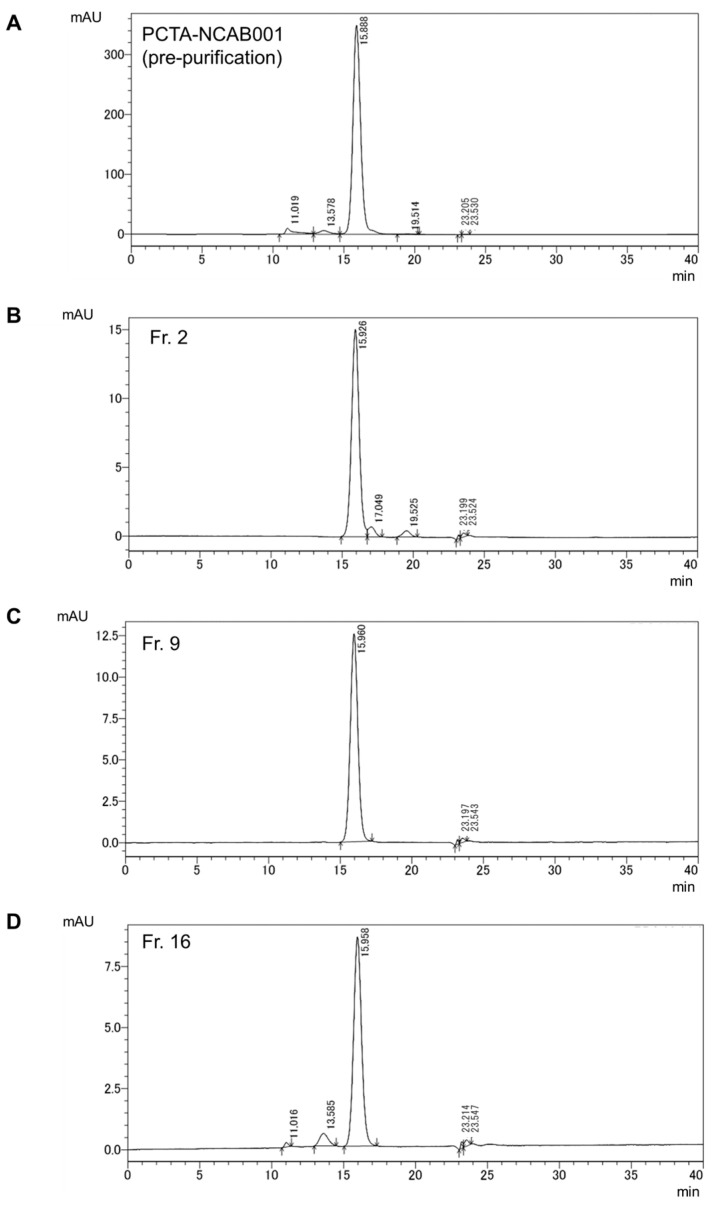
Size-exclusion chromatograms of PCTA-NCAB001 (**A**) and fractions 2 (**B**), 9 (**C**), and 16 (**D**) obtained from HIC. Monomeric PCTA-NCAB001 was eluted at the retention time of 16 min. Dimers and oligomers were found in PCTA-NCAB001 (**A**) and fraction 16 (**D**) at the retention times of 11 min and 13 min. Smaller size variants were found in PCTA-NCAB001 (**A**) and fraction 2 (**B**) at the retention times of 17 min and 19 min. These size variants were not detected in fraction 9 (**C**).

**Figure 4:**
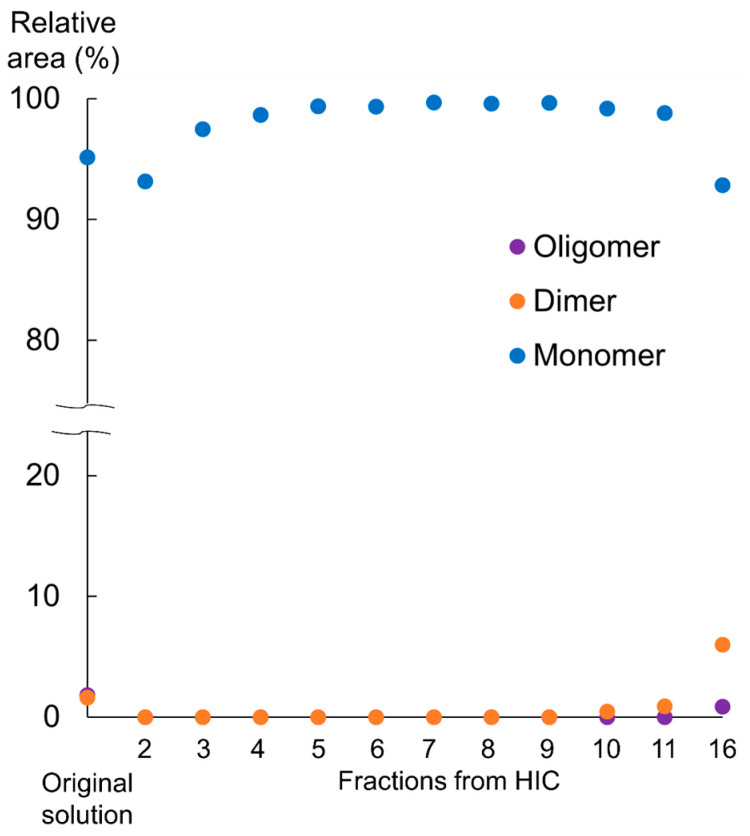
SEC-HPLC analysis of the fractions obtained from HIC. Relative peak area (%) of the oligomers (purple circle), dimers (orange circle), and monomers (blue circle) of PCTA-NCAB001 contained in the original solution and fractions obtained from HIC were plotted.

The size variants contained in PCTA-NCAB001 were removed by hydrophobic interaction chromatography (HIC). Combinations of mobile phases and columns for HIC are summarized in Table 2. The results of the HIC of PCTA-NCAB001, condition No. 1 in Table 2, are shown in Figure 2. The obtained fractions No. 2 to 11 and No. 16 were analyzed by SEC-HPLC. The fractions No. 12 to 15 did not have any specific absorbance at 280 nm; therefore, these fractions were not analyzed by SEC-HPLC. The representative chromatograms are shown in Figure 3. Fractions No. 2 to 9 did not contain dimer nor oligomer. These size variants were detected in fraction No. 16. The relative peak area of PCTA-NCAB001 and size variants are summarized in Figure 4 and Supplemental Table S1. Smaller size variants at retention times of 17 min and 19 min were found in fraction No. 2, but these variants were not found in fractions 3 through 11 nor 16.

The other conditions of the HIC, conditions No. 2 to 5 in Table 2, did not separate larger size variants from the PCTA-NCAB001 monomer. Under these conditions, both larger size variants and the monomer were not retained by the hydrophobic column. The anion exchange chromatography, conditions No. 6 to 8 in Table 2, also did not separate larger size variants from the monomer. With conditions No. 6, both larger size variants and the monomer were not retained by the anion exchange column. With condition No. 7 and 8, both larger size variants and the monomer were retained by the column, but separation by the salt gradient was not sufficient. The representative chromatograms obtained from purification conditions 2 to 8 are shown in Supplemental Figures S1–S6. The relative peak area (%) of PCTA-NCAB001 and size variants in purification conditions 2 to 8 are summarized in Supplemental Table S2.

### 2.3. Stability of PCTA-NCAB001 after the Removal of the Size Variants

**Figure 5:**
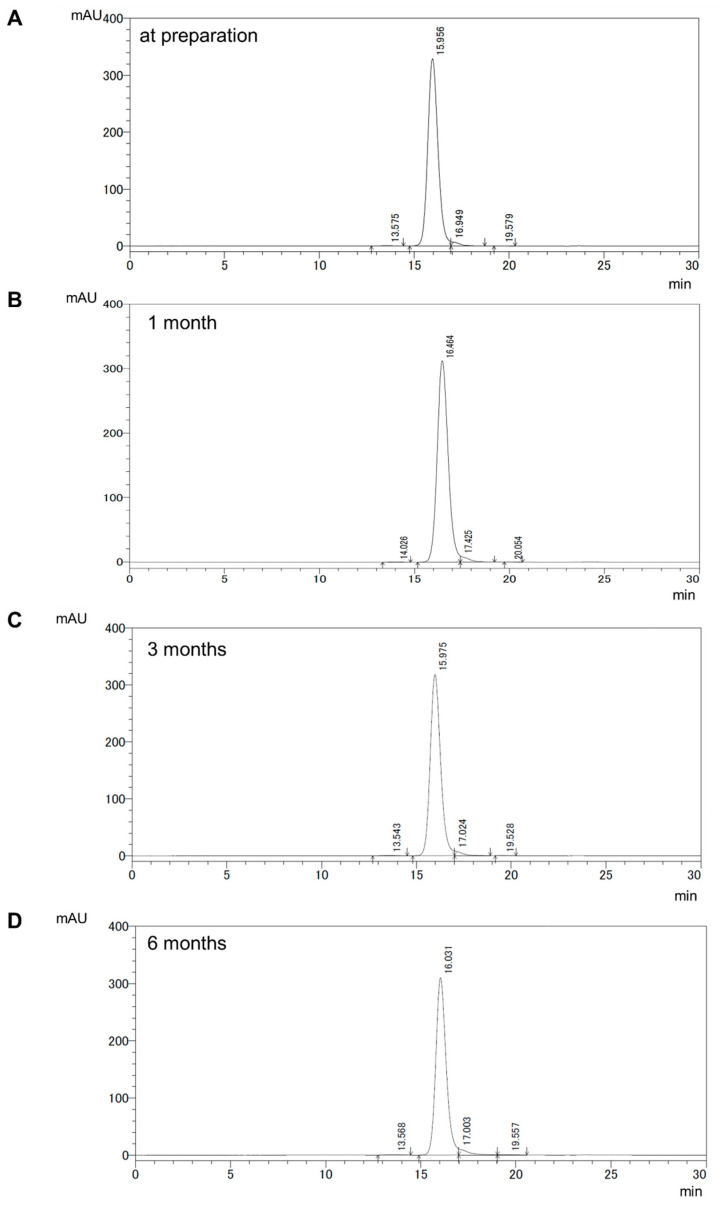
Size-exclusion chromatograms of PCTA-NCAB001 at the time of preparation (**A**), and one month (**B**), three months (**C**), and six months (**D**) of storage at 4 °C after the preparation.

Size variants contained in the PCTA-NCAB001 formulation at the time of the removal of the size variants were analyzed by SEC-HPLC. This sample was kept at 4 °C, and SEC-HPLC was performed at one, three, and six months after the preparation. The chromatograms are shown in Figure 5. Larger size variants, dimers at a retention time of 13 min and oligomers at a retention time of 11 min, were not detected at the time of preparation. Dimers gradually increased from 0.268% at one month to 0.531% at six months (Table 3). Oligomers were not detected throughout this storage period.

The samples were centrifuged, and pellets were not observed by visual inspection throughout the storage period. UV absorbance at 280 nm was measured before and after the centrifugation, and these values were not changed throughout the storage period (Table 4). These results support that the content of the insoluble impurity was not increased over the six months at 4 °C storage.

## 3. Discussion

The physicochemical properties of antibody–chelator conjugates should be carefully evaluated to ensure the quality of radiometal–antibody complexes as the radiopharmaceutical product. In this study, we determined the molecular weights of the larger size variants contained in PCTA-NCAB001 by SEC-MALS. Then, we developed a method to remove these size variants by HIC. Finally, the long-term stability of PCTA-NCAB001 after the removal of these size variants was confirmed.

We previously reported that a larger size variant was contained in NCAB001, and another larger size variant appeared in PCTA-NCAB001 through SEC-HPLC [16]. Since the contents of these larger size variants were too small, LC–MS was not sensitive enough to characterize them. As an alternative approach, we used SEC-MALS to determine the molecular weights of these variants in this study. SEC-MALS is a useful tool to investigate the aggregation and degradation of ADCs under various conditions [19,20,21,22]. ADCs and their size variants were separated using SEC-HPLC, and eluents were detected made with UV, RI, and MALS detectors online. The quantities and the molecular weights of macromolecules from a single injection can simultaneously be determined using commercially available and well-evaluated software [21]. Jiang et al. used the SEC-MALS system and reported that the aggregates generated during the preparation of gemtuzumab ozogamicin were predominantly composed of a mixture of dimers and oligomers [23].

By using this tool, we found that a larger size variant eluted at a retention time of 13 min in SEC had a molecular weight of 3.190 × 10^5^ g/mol (Figure 1 and Table 1). This value was twice that of the molecular weight of the main peak, 1.558 × 10^5^ g/mol, eluted at 16 min in SEC. The peak at the retention time of 13 min was found both in NCAB001 and PCTA-NCAB001 [16]. From these findings, we concluded that this larger size variant is the dimer of the antibody. We found another larger size variant eluted at a retention time of 11 min in the SEC of PCTA-NCAB001. MALS suggested that this peak contained several variants with an average molecular weight of 8.510 × 10^5^ g/mol. This exceeds 5 × 10^5^ g/mol, the exclusion limit of the SEC column (TSKgel G3000 SWXL), suggesting that trimers or even larger oligomers were eluted closely from this column. This interpretation was supported by the fact that the multi-variances of the oligomer, Mw/Mn and Mz/Mn, were larger than 1 (Table 1). This variant was not detected in NCAB001 antibody [16], suggesting that PCTA-NCAB001 formed complexed oligomers during antibody–chelator conjugation and the storage thereafter. An average of three molecules of PCTA was conjugated to the NCAB001 antibody [18]; therefore, oligomer formation by PCTA-NCAB001 was more complicated than that of conventional antibodies.

HIC has been used for the purification and characterization of proteins [24,25,26]. HIC separation uses mild conditions so that the conformations of proteins can be preserved [27]. Sodium chloride and sodium acetate have been used in previous works. In this work, HIC using sodium chloride and sodium acetate was adopted to remove the size variants in PCTA-NCAB001. The initial fraction contained smaller size variants with retention times of 17 min and 19 min (Figure 3B, fraction 2). The later fraction contained larger size variants, which were identified as dimer and oligomer by SEC-MALS in this study (Figure 3D and Figure 4, and Supplemental Table S1, fraction 16). Fractions 3 to 9 did not contain these size variants (Figure 3B and Table 3). By collecting fractions 3 to 9, the recovery yield of PCTA-NCAB001 was approximately 50%. Since we use 45 µg of PCTA-NCAB001 for a single patient dose, we decided that this yield was acceptable at this stage of process development. Fraction 2 of PCTA-NCAB001 contained smaller size variants (Figure 3B), which were not clearly detected in PCTA-NCAB001 (Figure 3A). The contents of these smaller size variants were too small to be detected even by MALS; however, they could be removed by HIC.

The mobile phase used in HIC to remove size variants (Table 2, No. 1) was the same as the formulation we developed to stabilize PCTA-NCAB001 and ^64^Cu-NCAB001 [18]. This brought us a manufacturing advantage in that only a protein concentration adjustment by a simple ultrafiltration step was needed after collecting the fractions containing the pure monomers of PCTA-NCAB001 from HIC. In this PCTA-NCAB001 product, the larger and smaller size variants were not detected (Figure 5A). The HIC system developed in this study can be applied to the development of other antibody derivatives, especially those with chelator ligands conjugated to the antibodies.

We stored the final product at 4 °C, and a trace amount of the dimer was detected at one month after the preparation (Figure 5B and Table 3). The amount of dimer increased gradually up to six months; but was still less than 1% (Figure 5C,D and Table 3). The oligomer was not detected within this storage period. The appearance of insoluble impurity was not suggested in PCTA-NCAB001 during the storage period by visual inspection, pellet formation after the centrifugation, or protein quantification. These findings support that PCTA-NCAB001 can be stored stably at 4 °C for six months after the removal of size variants. The radiolabeling of PCTA-NCAB001 with ^64^Cu was performed at 40 °C for 1 h. This labeling condition may not promote the additional formation of dimers and oligomers since (1) PCTA-NCAB001 was stable at 4 °C for six months, and (2) overnight conjugation of NCAB001 with p-SCN-Bn-PCTA at 37 °C formed substantially low amounts of the dimers and oligomers.

In our previous toxicity study [16], we used a formulation of the test substance containing approximately 5–6% of dimers and oligomers of the antibody. Our results suggested that their presence did not affect the safety of ^64^Cu-NCAB001. In this study, we could further develop the process to remove the size variants of PCTA-NCAB001. A total content of size variants below 5% as a specification for future products can be achieved by the methods developed herein. The long-term stability of PCTA-NCAB001 for more than one year is currently under investigation, and the relative binding potency of PCTA-NCAB001 against human recombinant EGFR after 1-year storage is being assessed. The sterility and endotoxin test results using ^64^Cu-NCAB001 prepared from PCTA-NCAB001 are also being evaluated.

## 4. Materials and Methods

### 4.1. Preparation of the PCTA-NCAB001 Formulation

The anti-EGFR antibody NCAB001 was prepared according to the cGMP at Mycenax Biotech Inc. (Jhunan, Taiwan), as previously reported [14]. NCAB001 antibody and the bifunctional chelator p-SCN-Bn-PCTA (Macrocyclics, Plano, TX, USA) were conjugated as previously reported [18]. Briefly, p-SCN-Bn-PCTA dissolved in dimethyl sulfoxide was added to the NCAB001 solution at a chelator-to-antibody molar ratio of 5:1. The mixtures were incubated overnight at 37 °C. After conjugation, the buffer was exchanged with PCTA-NCAB001 via ultrafiltration with 0.1 M acetate buffer (pH 6.0) containing 100 mM glycine and 76.3 μM polysorbate-80, and the concentration of PCTA-NCAB001 was adjusted to 2 mg/mL. The number of PCTA molecules conjugated to the antibody was determined using LC–MS. Approximately three PCTA molecules were conjugated to NCAB001.

### 4.2. Identification of the Size Variants Contained in PCTA-NCAB001 by SEC-MALS

The molecular weights of the size variants contained in the PCTA-NCAB001 conjugate were determined using SEC-MALS. An LC system (LC-20A, Shimadzu Corporation, Kyoto, Japan) and a TSKgel G3000 SWXL column (300 mm × 7.8 mm, 5 μm, Tosoh Bioscience, Tokyo, Japan) were used. The temperature of the column was maintained at 25 °C. Elution was conducted using a potassium phosphate buffer containing 250 mM potassium chloride at a flow rate of 0.5 mL/min and was monitored at a UV wavelength of 280 nm. The eluents from SEC were analyzed by MALS (DAWN HELEOS II, Wyatt Technology, Santa Barbara, CA, USA) and the Refractive Index Detector (Optilab T-rEX, Wyatt Technology), and molecular weights were calculated using the ASTRA software (version 7, Wyatt Technology) through the following equations [19,20,21,22].

(1) $${\left(\frac{Kc_{i}}{R_{\theta i}}\right)}^{1/2}={\left(\frac{1}{M_{i}P(\theta )}+2A_{2}c_{i}\right)}^{1/2}$$

$$\frac{1}{P(\theta )}=1+\left(\frac{1}{3}\right){\left(\frac{4\pi n_{0}}{\lambda }\right)}^{2}<S^{2}>\sin^{2}\left(\frac{\theta }{2}\right)$$

In this expression,

*K* is the optical constant 4π^2^ × *n*_0_^2^ × (d*n*/d*c*)^2^/(*λ*^4^ × *N*_0_).

*n*_0_ is the refractive index of the solvent.

*dn/dc* is the incremental of the refractive index.

*λ* is the vacuum wavelength of the laser.

*N*_0_ is Avogadro’s number.

*M_i_* is the molecular weight at retention time *i*.

*R_θi_* is the excess Rayleigh ratio at retention time *i* as a function of scattering angle θ.

*A_2_* is the second virial coefficient in the virial expansion of the osmotic pressure.

*c_i_* is the solute concentration at retention time *i*.

*P(θ)* describes the angular dependence of the scattered light.

<*S*^2^>^1/2^ is the mean square radius.

The concentrations of eluents were sparse, and *2A_2_c_i_* is small enough to 1/MiP(*θ*); therefore, Equation (1) can be proximate to the following equation:

(2) $${\left(\frac{Kc_{i}}{{R_{\theta }}_{i}}\right)}_{c=0}^{1/2}=\frac{1}{{M_{i}}^{1/2}}\left(1+\frac{1}{6}{\left(\frac{4\pi n_{0}}{\lambda }\right)}^{2}<S^{2}>\sin^{2}(\frac{\theta }{2})\right)$$

where *R_θi_* and *c_i_* were measured by SEC-MALS. Using these values, *sin^2^(θ/2)* and *(Kc_i_/R_θi_)*^1/2^ were plotted (Zimm plot), and the molecular weight of each retention time Mi was determined by proximation of *θ* to zero. As *dn/dc*, 0.185 mL/g was used in this study since this value is commonly used as the standard protein. BSA was measured to calibrate the MALS system. The injected volume to the SEC-MALS system was 100 μL for BSA and 40 μL for PCTA-NCAB001. The protein concentration of the injected sample was 2.0 mg/mL.

### 4.3. Removal of the Size Variants Contained in PCTA-NCAB001 by HIC

The size variants contained in PCTA-NCAB001 were removed by HIC. Five mL of 2 mg/mL PCTA-NCAB001 solution was loaded into the hydrophobic column (TOYOPERL Phenyl FT-750F, 1.0 × 6.0 cm, column volume 4.7 mL, TOSO Bioscience, Tokyo, Japan). 0.1 M acetate buffer (pH 6.0) containing 100 mM glycine and 76.3 μM polysorbate-80 was used as mobile phase A, and mobile phase A with 1 M NaCl was used as mobile phase B. The flow rate was 2.5 mL/min, and eluents were monitored by UV at 280 nm. 50 mL of mobile phase A was applied to the column, followed by a linear gradient of mobile phase B from 0% to 100% in 40 mL. The eluents were fractionated in 5 mL each. The obtained fractions were analyzed by SEC-HPLC as reported previously [16].

Some additional mobile phases were used with the hydrophobic column for comparison. The anion exchange chromatography column (HiTrap Q FF, 1.6 × 2.5 cm, column volume 5 mL, Cytiva, Marlborough, MA, USA) was also used for comparison. Combinations of mobile phases and columns are summarized in Table 2. In conditions No. 2 to 5, the hydrophobic column Phenyl FT-750F was used as condition No. 1. The sample was introduced into the column equilibrated at a high salt concentration, aiming to recover the monomer in the flow-through fractions and elute the oligomers at a lower salt concentration. The conductance of the injected sample was increased in condition No. 2 and decreased in condition No. 3 and 4. In condition No. 5, glycine was removed from the injected samples. In conditions No. 6 to 8 using HiTrap Q FF, the sample was diluted and introduced into the column equilibrated at a low salt concentration. We aimed to collect the monomers in the flow-through fraction and elute the oligomers by increasing the salt concentration.

### 4.4. Stability of PCTA-NCAB001 after Removal of the Size Variants

Fractions from 3 to 9 obtained by HIC (Figure 2) were collected, mixed, and concentrated with an ultrafiltration filter (Amicon Ultra-15, molecular cutoff 50 kDa, cellulose membrane, Merck KGaA, Darmstadt, Germany). Protein concentration was adjusted as 2.21 mg/mL with 0.1 M acetate buffer (pH 6.0) containing 100 mM glycine and 76.3 μM polysorbate-80 and stored at 4 °C for six months. The stability of PCTA-NCAB001 after the removal of the size variants was evaluated by SEC-HPLC at the time of preparation and at one, three, and six months after the preparation. To evaluate the content of the insoluble impurity, samples were centrifuged (15,000× *g*, 10 min, 4 °C) and the pellets were observed by visual inspection. The protein concentrations of the supernatants were measured by UV absorbance at 280 nm and compared with that of the solution before centrifugation.

## 5. Conclusions

Dimers and oligomers were identified by SEC-MALS in the antibody–chelator conjugate PCTA-NCAB001. These larger size variants, together with some smaller size variants, could be removed by HIC. The PCTA-NCAB001 product, after the removal of these size variants, remains stable when stored at 4 °C for six months. These results, together with our previous findings, will be used to assess the quality of PCTA-NCAB001 as the final intermediate of ^64^Cu-NCAB001 for the PET imaging of pancreatic cancer. The methods developed here can be applied to the development of other antibody–radiometal conjugates for the PET and radioimmunotherapy of malignant cancers.

## Figures and Tables

**Table 1:** Molecular weights of PCTA-NCAB001 and the size variants.

Sample		Number-Mean M.W. Mn * (g/mol)	Mass-Mean M.W. Mw * (g/mol)	Z-Mean M.W. Mz * (g/mol)	Multi-Variance Mw/Mn	Multi-Variance Mw/Mz
BSA	Monomer	6.766 × 10^4^	6.770 × 10^4^	6.775 × 10^4^	1.001	1.001
PCTA-NCAB001	Monomer	1.558 × 10^5^	1.559 × 10^5^	1.560 × 10^5^	1.000	1.001
	Dimer	3.190 × 10^5^	3.195 × 10^5^	3.201 × 10^5^	1.002	1.003
	Oligomer	8.510 × 10^5^	9.720 × 10^5^	1.174 × 10^6^	1.142	1.380

M.W.: molecular weight; * absolute molecular weight; Mn = ∑(*N_i_·M_i_*)/∑(*Ni*); Mw = ∑(*N_i_·M_i_*^2^)/∑(*Ni·M_i_*); Mz = ∑(*N_i_·M_i_*^3^)/∑(*N_i_·M_i_*^2^).

**Table 2:** Columns and mobile phases used for the removal of size variants from PCTA-NCAB001.

No.	Column	Mobile Phase A	Mobile Phase B	Injected Sample
1	Phenyl FT-750F	0.1 M acetate buffer (pH 6.0) containing 100 mM glycine and 76.3 μM polysorbate-80	Mobile phase A + 1 M NaCl	PCTA-NCAB001 *
2		0.1 M phosphate buffer (pH 6.8)	Mobile phase A + 150 mM sodium sulphate	PCTA-NCAB001 * + 150 mM sodium sulphate
3		50 mM acetate buffer (pH 6.0)	Mobile phase A + 50 mM glycine	PCTA-NCAB001 * × 2 diluted with H_2_O
4		0.1 M acetate buffer (pH 6.0)	Mobile phase A + 100 mM glycine	PCTA-NCAB001 *
5		0.3 M acetate buffer (pH 6.0)	50 mM acetate buffer (pH 6.0)	PCTA-NCAB001 50 kDa filtrated
6	HiTrap Q FF	0.1 M acetate buffer (pH 6.0) containing 100 mM glycine and 76.3 μM polysorbate-80	Mobile phase A + 1 M NaCl	PCTA-NCAB001 *
7		50 mM Tris/HCl (pH 8.0)	Mobile phase A + 1 M NaCl	PCTA-NCAB001 * × 2 diluted with mobile phase A
8		50 mM Tris/HCl (pH 8.0)	Mobile phase A + 1 M NaCl	PCTA-NCAB001 * × 3 diluted with mobile phase A

* A concentration of 2 mg/mL PCTA-NCAB001 in 0.1 M acetate buffer (pH 6.0) containing 100 mM glycine and 76.3 μM polysorbate-80, 5 mL.

**Table 3:** SEC-HPLC analysis of PCTA-NCAB001 after the removal of size variants.

	Oligomer		Dimer		Monomer	
	Peak Area	Area (%)	Peak Area	Area (%)	Peak Area	Area (%)
At preparation	N.D.	N.D.	N.D.	N.D.	12811561	100.00
1 month	N.D.	N.D.	35130	0.268	12865058	99.732
2 months	N.D.	N.D.	59230	0.454	12716827	99.546
6 months	N.D.	N.D.	69391	0.531	12478288	99.469

N.D.: not detected.

**Table 4:** Insoluble impurity contained in the PCTA-NCAB001 formulation after the removal of size variants.

	Protein Concentration (mg/mL)		Pellet after Centrifugation
	Before Centrifugation	After Centrifugation	
At preparation	2.21	2.24	N.D.
1 month	2.27	2.26	N.D.
2 months	2.22	2.29	N.D.
6 months	2.21	2.22	N.D.

N.D.: not visually detected.

## Data Availability

Data is contained within the article and supplementary material.

## Supplementary Materials

The following supporting information can be downloaded at: https://www.mdpi.com/article/10.3390/ph16101341/s1, Figure S1: Hydrophobic interaction chromatogram (A) and size exclusion chromatogram of PCTA-NCAB001 (B) under condition 2; Figure S2: Hydrophobic interaction chromatogram (A) and size exclusion chromatogram of PCTA-NCAB001 (B) under condition 3; Supplementary Figure S3: Hydrophobic interaction chromatogram (A) and size exclusion chromatogram of PCTA-NCAB001 (B) under condition 4; Supplementary Figure S4: Hydrophobic interaction chromatogram (A) and size exclusion chromatogram of PCTA-NCAB001 (B) under condition 5; Supplementary Figure S5: Anion exchange chromatogram (A) and size exclusion chromatogram of PCTA-NCAB001 (B) under condition 6; Supplementary Figure S6: Anion exchange chromatogram of PCTA-NCAB001 under condition 7 (A) and condition 8 (B); Table S1: SEC-HPLC analysis of the fractions obtained from HIC (condition 1); Table S2: SEC-HPLC analysis of the flow-through fractions obtained from conditions 2 to 6.
