# Supplementary material for: Process to Remove the Size Variants Contained in the Antibody–Chelator Complex PCTA-NCAB001 for Radiolabeling with Copper-64
Source: Pharmaceuticals (Basel). 2023 Sep 22;16(10):1341. doi: 10.3390/ph16101341 (PMC10610008; doi:10.3390/ph16101341)
Supplement: Supplementary file 1 [file pharmaceuticals-16-01341-s001.zip › pharmaceuticals-2596993-SI.pdf]

Supplemental Information

The process to remove the size variants contained in the antibody-chelator complex PCTA-NCAB001 for the radiolabeling with copper-64

Yukie Yoshii, Hiroki Matsumoto, Chika Igarashi, Tomoko Tachibana, Fukiko Hihara, Mitsuhiro Shinada, Atsuo Waki, Sei Yoshida, Kenichiro Naito, Kimiteru Ito, Tatsuya Higashi, Hiroaki Kurihara and Makoto Ueno

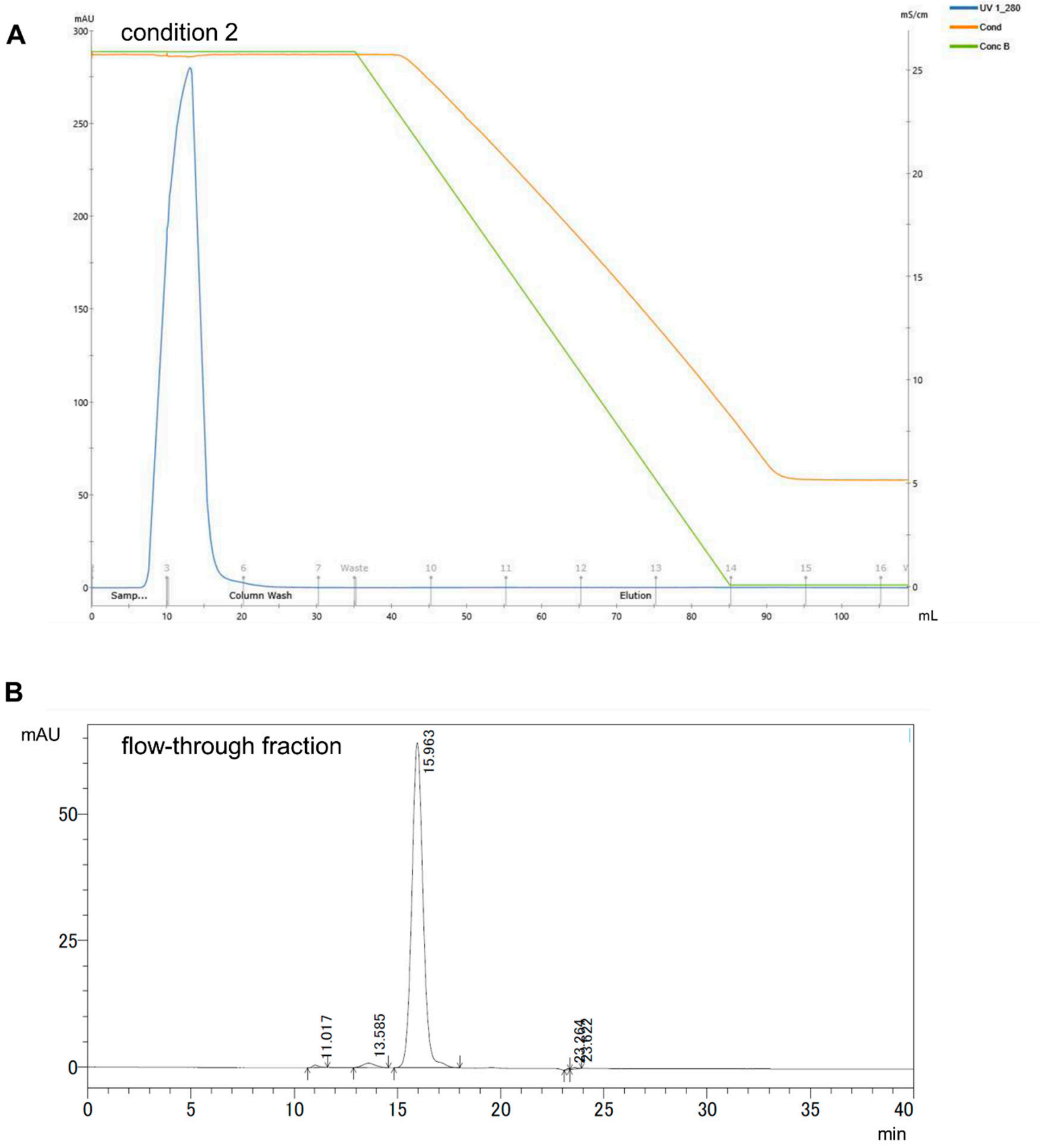

Supplementary Figure S1. Hydrophobic interaction chromatogram (A) and size exclusion chromatogram of PCTA-NCAB001 (B) under condition 2. Flow-through fraction (fraction 5) obtained from HIC (A) was analyzed by SEC-HPLC (B). Under this condition, both larger size variants and the monomer were not retained by the hydrophobic column.

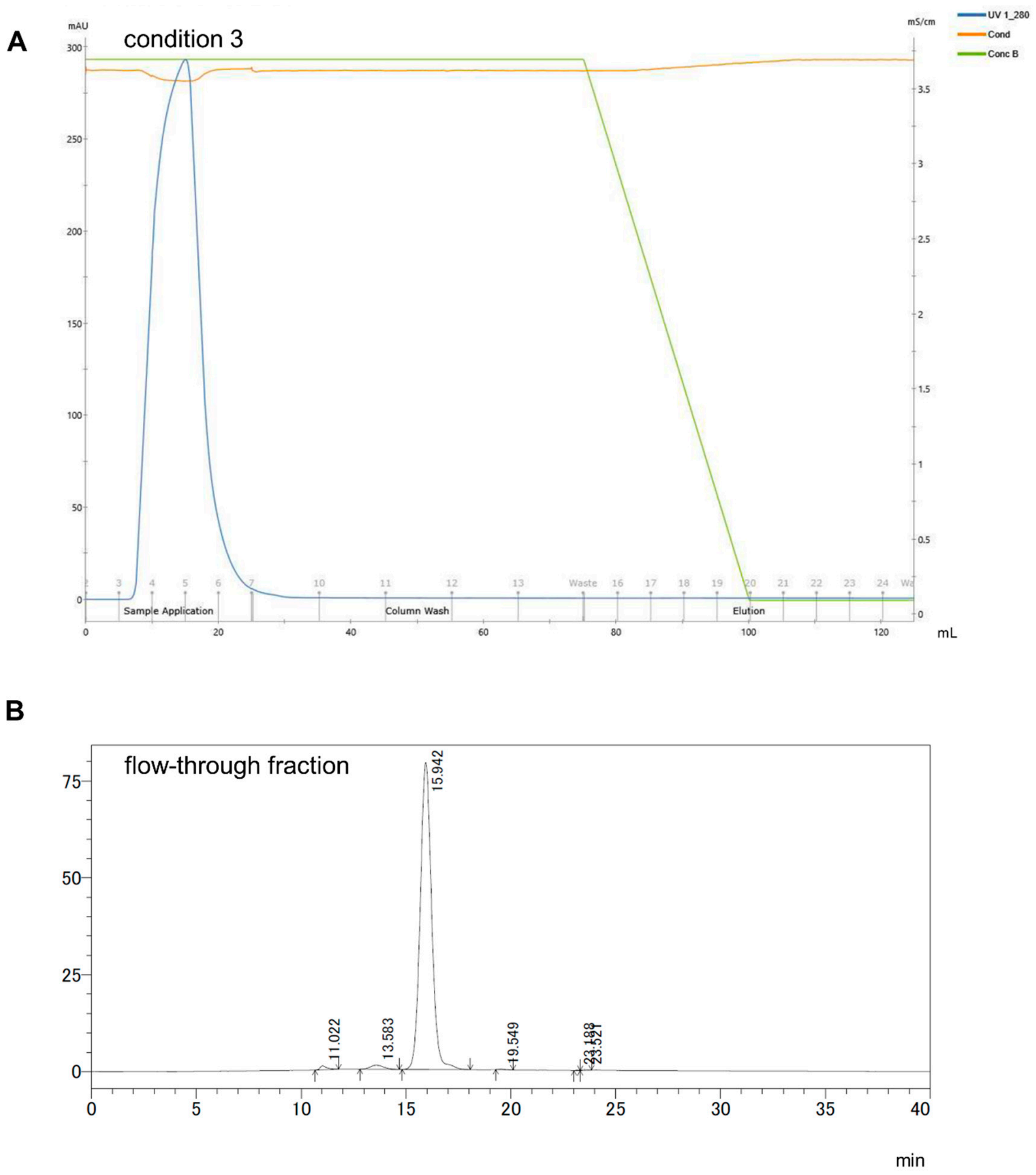

Supplementary Figure S2. Hydrophobic interaction chromatogram (A) and size exclusion chromatogram of PCTA-NCAB001 (B) under condition 3. Flow-through fraction (fraction 5) obtained from HIC (A) was analyzed by SEC-HPLC (B). Under this condition, both larger size variants and the monomer were not retained by the hydrophobic column.

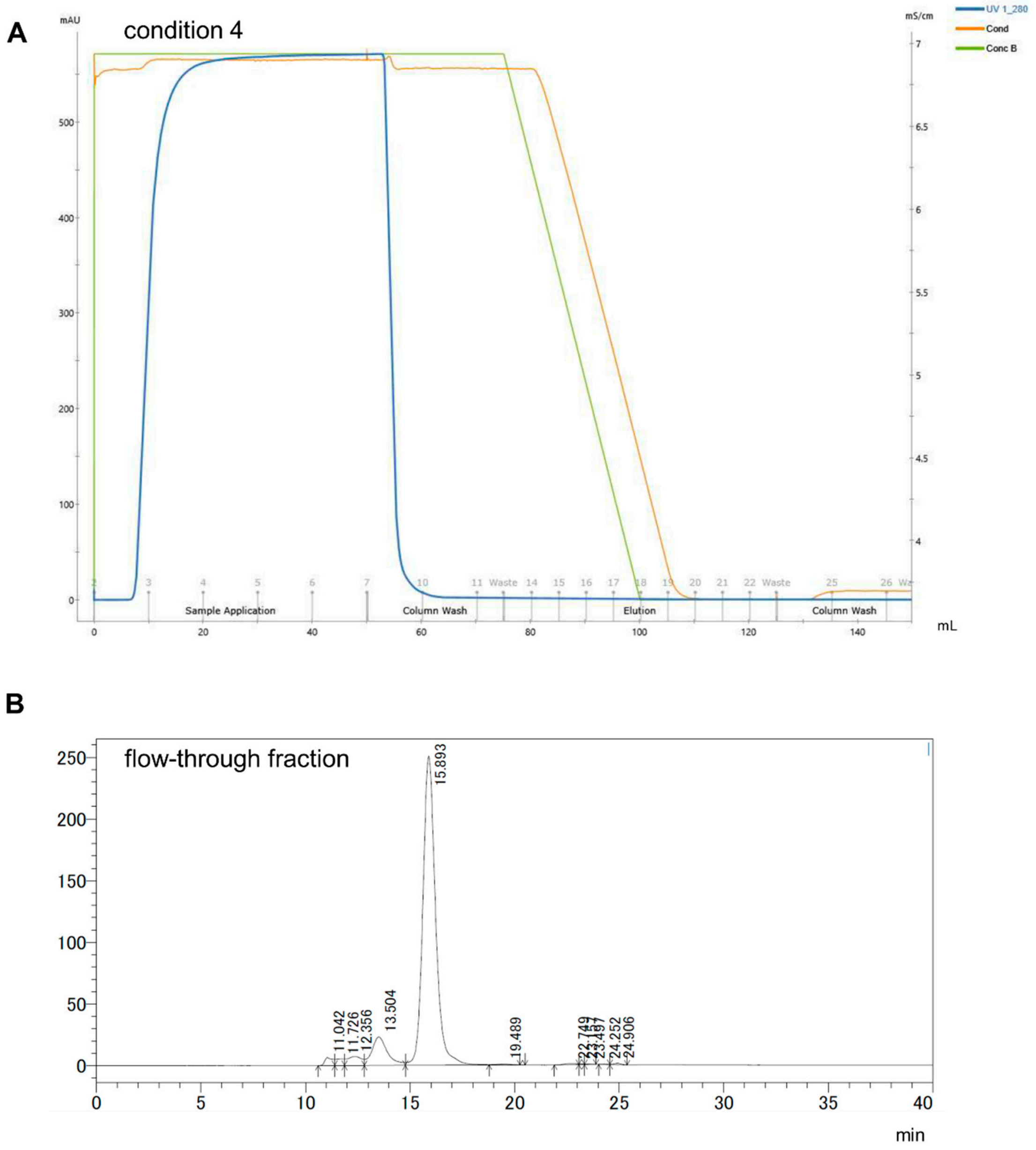

Supplementary Figure S3. Hydrophobic interaction chromatogram (A) and size exclusion chromatogram of P[illegible] NCAB001 (B) under condition 4. Flow-through fraction (fraction 5) obtained from HIC (A) was analyzed by SE[illegible] HPLC (B). Under this condition, both larger size variants and the monomer were not retained by the hydropho[illegible] column.

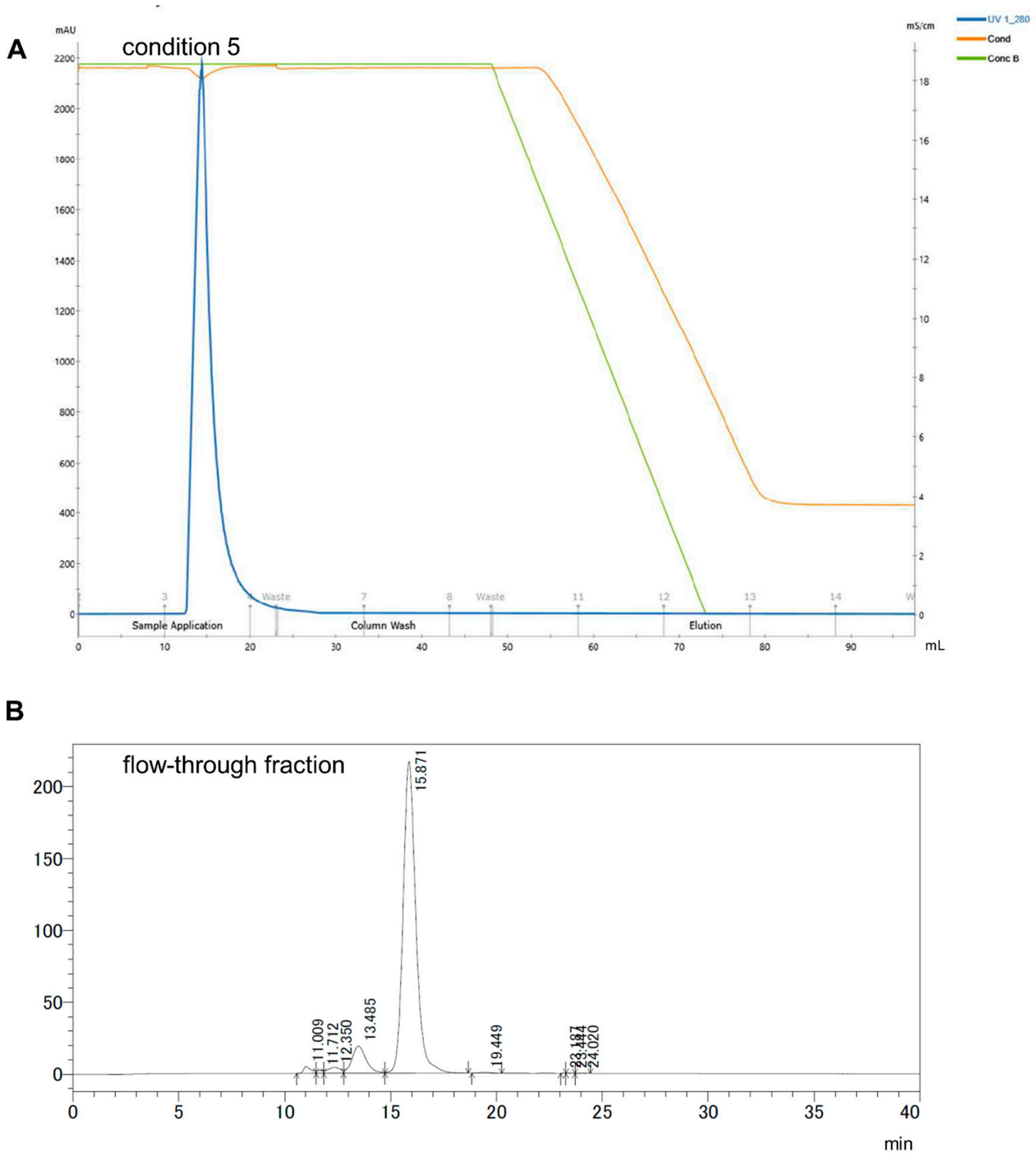

Supplementary Figure S4. Hydrophobic interaction chromatogram (A) and size exclusion chromatogram of PCTA-NCAB001 (B) under condition 5. Flow-through fraction (fraction 3) obtained from HIC (A) was analyzed by SEC-HPLC (B). Under this condition, both larger size variants and the monomer were not retained by the hydrophobic column.

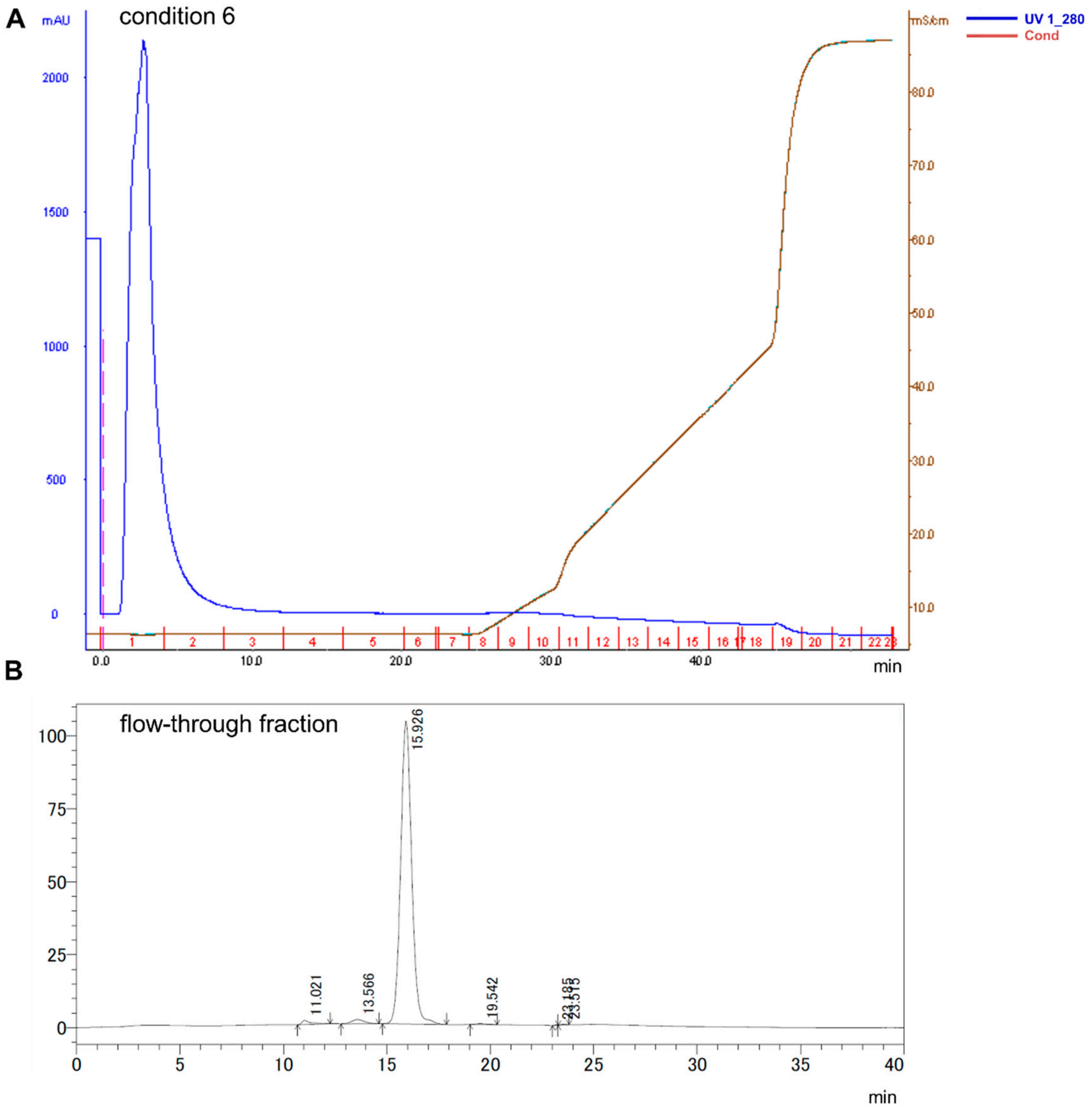

Supplementary Figure S5. Anion exchange chromatogram (A) and size exclusion chromatogram of PCTA-NCAB001 (B) under condition 6. Flow-through fraction (fraction 1) obtained from anion exchange chromatogram (A) was analyzed by SEC-HPLC (B). Under this condition, both larger size variants and the monomer were not retained by the anion exchange column.

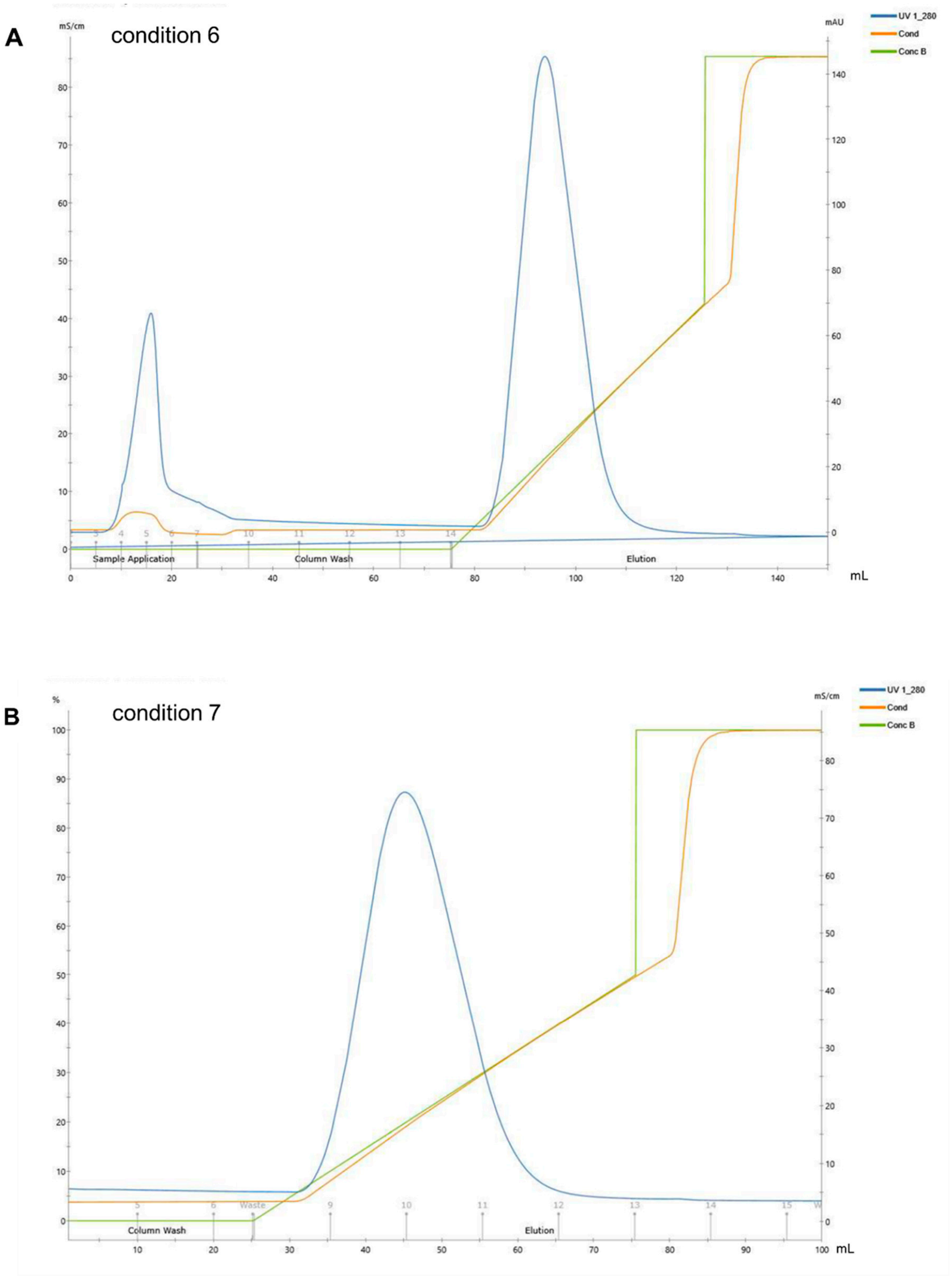

Supplementary Figure S6. Anion exchange chromatogram of PCTA-NCAB001 under condition 7 (A) and condition 8 (B). Under these conditions, both larger size variants and the monomer were retained by the column, but separation by the salt gradient was not sufficient.

Supplementary Table S1. SEC-HPLC analysis of the fractions obtained from HIC (condition 1)

	**Oligomer**		**Dimer**		**Monomer**	
	**Peak area**	**Area (%)**	Peak area	Area (%)	Peak area	Area (%)
Original solution	248771	1.825	218832	1.606	12966137	95.136
Fr.2	N.D.	N.D.	N.D.	N.D.	563283	93.166
Fr.3	N.D.	N.D.	N.D.	N.D.	1756706	97.470
Fr.4	N.D.	N.D.	N.D.	N.D.	1188814	98.669
Fr.5	N.D.	N.D.	N.D.	N.D.	870654	99.369
Fr.6	N.D.	N.D.	N.D.	N.D.	697108	99.349
Fr.7	N.D.	N.D.	N.D.	N.D.	603162	99.688
Fr.8	N.D.	N.D.	N.D.	N.D.	516809	99.601
Fr.9	N.D.	N.D.	N.D.	N.D.	461388	99.657
Fr.10	N.D.	N.D.	1900	0.444	424027	99.180
Fr.11	N.D.	N.D.	3713	0.894	410360	98.798
Fr.16	3091	0.870	21309	5.995	330027	92.851

N.D.: not detected.

Supplementary Table S2. SEC-HPLC analysis of the flow-through fractions obtained from conditions 2 to 6

	**Oligomer**		**Dimer**		**Monomer**	
	**Peak area**	**Area (%)**	Peak area	Area (%)	Peak area	Area (%)
Condition 2	10719	0.431	37367	1.300	2431026	97.747
Condition 3	22971	0.749	48640	1.585	2988066	97.368
Condition 4*	657123	5.255	1189089	9.509	10548177	84.356
Condition 5*	336871	3.273	875792	8.509	9046491	87.893
Condition 6	42562	1.070	65168	1.638	3853431	96.836

*Conditions 4 and 5: Area of the oligomer is sum of three peaks with retention time from 11 to 13 min.
